# Radiomic analysis of Gd-EOB-DTPA-enhanced MRI predicts Ki-67 expression in hepatocellular carcinoma

**DOI:** 10.1186/s12880-021-00633-0

**Published:** 2021-06-15

**Authors:** Yanfen Fan, Yixing Yu, Ximing Wang, Mengjie Hu, Chunhong Hu

**Affiliations:** 1 Department of Radiology, The First Affiliated Hospital of Soochow University, Shizi Street 188, Suzhou, 215006 Jiangsu People’s Republic of China; 2 Institute of Medical Imaging of Soochow University, Shizi Street 188, Suzhou, 215006 Jiangsu People’s Republic of China

**Keywords:** Hepatocellular carcinoma, Vessels encapsulating tumor clusters, Enhanced MRI, Gd-EOB-DTPA, Radiomics

## Abstract

**Background:**

Nuclear protein Ki-67 indicates the status of cell proliferation and has been regarded as an attractive biomarker for the prognosis of HCC. The aim of this study is to investigate which radiomics model derived from different sequences and phases of gadolinium-ethoxybenzyl-diethylenetriamine pentaacetic acid (Gd-EOB-DTPA)-enhanced MRI was superior to predict Ki-67 expression in hepatocellular carcinoma (HCC), then further to validate the optimal model for preoperative prediction of Ki-67 expression in HCC.

**Methods:**

This retrospective study included 151 (training cohort: n = 103; validation cohort: n = 48) pathologically confirmed HCC patients. Radiomics features were extracted from the artery phase (AP), portal venous phase (PVP), hepatobiliary phase (HBP), and T2-weighted (T2W) images. A logistic regression with the least absolute shrinkage and selection operator (LASSO) regularization was used to select features to build a radiomics score (Rad-score). A final combined model including the optimal Rad-score and clinical risk factors was established. Receiver operating characteristic (ROC) curve analysis, Delong test and calibration curve were used to assess the predictive performance of the combined model. Decision cure analysis (DCA) was used to evaluate the clinical utility.

**Results:**

The AP radiomics model with higher decision curve indicating added more net benefit, gave a better predictive performance than the HBP and T2W radiomic models. The combined model (AUC = 0.922 vs. 0.863) including AP Rad-score and serum AFP levels improved the predictive performance more than the AP radiomics model (AUC = 0.873 vs. 0.813) in the training and validation cohort. Calibration curve of the combined model showed a good agreement between the predicted and the actual probability. DCA of the validation cohort revealed that at a range threshold probability of 30–60%, the combined model added more net benefit compared with the AP radiomics model.

**Conclusions:**

A combined model including AP Rad-score and serum AFP levels based on enhanced MRI can preoperatively predict Ki-67 expression in HCC.

**Supplementary Information:**

The online version contains supplementary material available at 10.1186/s12880-021-00633-0.

## Background

Hepatocellular carcinoma (HCC) remains a leading cause of cancer-related morbidity and mortality worldwide, which accounts for 75–85% of all primary liver cancer cases [1]. The poor prognosis of HCC after surgical resection is mainly due to recurrence and metastasis [2, 3]. Nuclear protein Ki-67 expression level- indicates the status of cell proliferation activity which corresponds with tumor biological behavior, treatment efficacy and prognosis [4, 5]. Previous studies have demonstrated that high Ki-67 expression was associated with poor overall survival (OS) [6–10], disease-free survival (DFS) [6, 9, 11], relapse-free survival (RFS) [8, 9, 12]. In particular, Ki-67 is proposed to be an attractive therapeutic target for cancer because it is highly expressed in most malignant cells but rarely detected in normal cells, though this targeting Ki-67 therapy has not been applied in the clinical [5]. Accurate identification of Ki-67 expression level is crucial for prognosis and treatment decision-making to achieve a satisfactory outcome. However, it is difficult to differentiate the nuances among HCCs with different Ki-67 expression through conventional imaging.

Current radiomics, which involves numerous advanced, quantitative, high-throughput features extracted from medical images, has been used to develop diagnostic, predictive, and prognostic models [13, 14]. Previous studies have reported that tumor characteristics at the cellular and genetic levels can be reflected in the phenotypic patterns and subsequently captured by radiomics signatures [15–20]. Gadolinium ethoxybenzyl-diethylenetriamine pentaacetic acid (Gd-EOB-DTPA), which has characteristics of both a blood-pool agent and a hepatobiliary agent, is commonly used in clinical practice. Previous studies have applied texture analysis on Gd-EOB-DTPA-enhanced MRI to preoperatively predict Ki-67 expression in patients with HCC and indicated that the texture analysis was superior to subjective MRI characteristics determined by radiologists and obtained a good result in predicting Ki-67 expression [21, 22]. Although previous studies were valuable, they have not compared predictive performance of radiomics models derived from different sequences and phases based on Gd-EOB-DTPA-enhanced MRI.

Thus, this study aimed to develop and compare predictive performance of radiomics models derived from different sequences and phases based on Gd-EOB-DTPA-enhanced MRI, then to further validate the optimal model for preoperative prediction of Ki-67 expression in patients with HCC.

## Methods

### Patients

This is a retrospective study for which ethical approval was obtained and informed consent from patients was waived. Between January 2013 and November 2019, patients who underwent Gd-EOB-DTPA-enhanced MRI examination before surgery or biopsy were consecutively included in this study according to the following inclusion and exclusion criteria. The inclusion criteria were: (1) pathologically confirmed HCC; (2) received Gd-EOB-DTPA-enhanced MRI of the liver within 1 month before surgery or biopsy; (3) images without obvious artifact; (4) if multiple lesions were present, the largest one was selected with matched  pathological and immunohistochemical diagnosis. The exclusion criteria were: (1) received previous treatment, such as anti-tumor therapies, radiofrequency ablation, transcatheter arterial chemoembolization (TACE), and so on; (2) incomplete clinical or pathological information. All enrolled patients were randomly divided into training and validation cohorts at a ratio 7:3.

### Histopathological examination

The tumor tissue sections were stained using monoclonal mouse anti-human Ki-67 antibody (Beijing Zhongshan Golden Bridge Biotechnology Company, Beijing, China). The Ki-67 expression was evaluated by calculating the frequency of 1 Ki-67-positive cells. Ki-67 was considered positive when the cell nuclei were stained brown yellow. Immunoreactive cells were classified as low Ki-67 expression (≤ 14% immune-reactivity) or high Ki-67 expression (> 14% immune-reactivity) according to previous studies [5, 16]. Referring to previous study, we dichotomized histologic subtypes using low-grade tumors and high-grade tumors. Low-grade tumors correspond to well differentiated, well and moderately differentiated, and moderately differentiated HCC. High-grade tumors correspond to moderately and poorly differentiated, poorly differentiated, and undifferentiated HCC.

### MRI protocol

The details of MRI protocol and the sequences used in this study were presented in the Additional file 1.

### Tumor segmentation

Tumor segmentation was manually performed on (arterial phase, AP), (portal venous phase, PVP), (Hepatobiliary phase, HBP) and T2W images with 3D Slicer (http://www.slicer.org), and a three-dimensional (3D) region of interest (ROI) that covered the whole tumor was delineated along the border of tumors. HBP or T2W images were first for manual segmentation. Subsequently, AP and PVP images were delineated, as the tumor margins on HBP or T2W images were clearer than that on AP and PVP images. Taking this delineating order would mitigate software-related segmentation errors. The segmentation was independently performed by two radiologists (Y.Y., 10 years of liver imaging experience; Y.F., 8 years of liver imaging experience) in 30 randomly chosen patients to assess inter-observe reproducibility. The segmentation was performed again by the radiologist (Y.F.) at another day to assess the intra-observe reproducibility. The remaining images of patients were segmented by the radiologist (Y.F.). Both radiologists were blinded to the clinical outcomes.

### Preprocessing and radiomic features extraction

Before radiomic features extraction, preprocessing of images was performed, including Laplacian of Gaussian (LoG) preprocessing, wavelet transformations, bin discretization and radiomic matrix symmetry. Features extraction was performed using the Slicer Radiomics extension, which incorporates the PyRadiomics library into 3D Slicer [23]. Extracted features included first order statistics, shape and texture features, which were gray level co-occurrence matrix (GLCM), gray level size zone matrix (GLSZM), gray level run length matrix (GLRLM), gray level dependence matrix (GLDM) and neighboring gray tone dependence matrix (NGTDM). Among these features, flatness and least axis from shape features were excluded based on the definition of the feature, as discussed in the documentation of PyRadiomics, and sum average was excluded because it is directly correlated with joint average [24]. Thus, a total of 1,300 radiomic features were extracted for each unique lesion.

### Radiomic feature selection and model development

The least absolute shrinkage and selection operator (LASSO) logistic regression with 5-fold cross-validation was used to select the most useful features in the training cohort. Rad-score was calculated for each patient using the linear combination of selected features multiplied by their respective coefficients.

### Comparison of radiomics model in the training and validation cohort

These models assessed in the training cohorts were applied to validation cohorts. The Receiver operating characteristic (ROC) curve, Delong test, calibration curve and decision curve analysis (DCA) were utilized to illustrate the diagnostic performances of these constructed models, and the cutoff values were selected according to the Youden index to determine the corresponding sensitivity and specificity.

## Combined model development and validation

For the development of combined model, we performed multivariate logistic regression analysis of clinical factors in training cohort, including age, sex, hepatitis B, hepatitis C, cirrhosis, serum alanine aminotransferase (ALT) level, serum aspartate aminotransferase (AST) level, serum gamma-glutamyl transferase (GGT) level, and serum alpha-fetoprotein (AFP) level. Clinical factors that reached statistical significance with *P* values less than 0.05 were selected into the combined model, which also included the optimal Rad-score.

Calibration curves were adopted to analyze the diagnostic performance of the combined model in both training and validation cohort. Decision curve analysis was conducted to determine the clinical usefulness of the combined model by quantifying the net benefits at different threshold probabilities in the validation cohort.

### Statistical analysis

The continuous variables were described as median and interquartile range, and the categorical variables were described as frequency and percentage. D’Agostino–Pearson test was used to test normality of dates. Independent sample t-test or Mann–Whitney U nonparametric rank sum test was used to compare clinical characteristics between the training and validation cohort, and between high Ki-67 expression and low Ki-67 expression groups in the training and validation cohort for continuous variables, while.

the Chi-squared test or Fisher exact test were conducted for categorical variables. Two-sided *P* values < 0.05 were considered statistically significant. The inter-observer and the intra-observer reproducibility to the extracted features were assessed by the intra-class correlation coefficient (ICC). ICC ≥ 0.8, 0.5–0.79 and < 0.5 indicated high, middle, and low consistency, respectively [25]. LASSO logistic regression, and multivariable logistic regression analysis were performed to select radiomics features and clinical risk factors using the “glmnet” and “rms” package running in R software, version 3.0.1 (http://www.Rproject.org.org). The calibration and decision curve were plotted using the “rms” and “rmda” package. Other statistical analyses were performed using the MedCalc software (Version 16.2.0, https://www.medcalc.org).

## Results

### Baseline characteristics

One hundred fifty-one patients were collected, including 103 patients in the training cohort and 48 patients in the validation cohort (Table 1). Baseline characteristics were not significantly different between training and validation cohort. Among all 151 patients, high Ki-67 expression was pathologically diagnosed in 112 patients (74.2%), low Ki-67 expression was pathologically diagnosed in 39 patients (25.8%). In both cohorts, the serum AFP levels and tumor grade were significantly higher in high Ki-67 expression group than that in low Ki-67 expression group. In both cohorts, low-grade tumors were more frequently in patients with low Ki-67 expression group. In the training cohort, the number of patients with hepatitis B in high Ki-67 expression was larger than that in the low Ki-67 expression group (Table 2).

**Table 1:** Baseline clinical characteristics of the training and validation cohort

	Total (n = 151)	Training (n = 103)	Validation (n = 48)	*P* values
Age(years), median (IQR)	58.0 (51.0, 67.0)	61.0 (50.3, 68.0)	56.0 (51.0, 64.0)	0.307
Gender, no.(%)				0.355
Male	119 (78.8)	79 (76.7)	40 (83.3)	
Female	32 (21.2)	24 (23.3)	8(16.7)	
ALT, (U/L), median (IQR)	33.5 (20.8, 47.8)	29.70 (20.7, 45.3)	35.4 (20.9, 52.9)	0.220
AST, (U/L), median (IQR)	33.0 (24.4, 44.1)	32.4 (24.5, 43.4)	33.9 (24.0, 49.8)	0.407
GGT, (U/L), median (IQR)	50.8 (29.2, 108.8)	49.6 (30.2, 92.7)	52.1 (27.9, 144.5)	0.771
AFP, (µg/L), median(IQR)	15.3 (3.1, 417.5)	15.5 (3.1, 527.4)	11.6 (2.9, 301.6)	0.718
AFP group.no(%)				0.555
≤ 20 µg/L	79 (52.3)	54 (52.4)	25 (52.1)	
20–400 µg/L	34 (22.5)	21 (20.4)	13 (27.1)	
> 400 µg/L	38 (25.2)	28 (27.2)	10 (20.8)	
Hepatitis B, no.(%)				0.856
Negative	36 (23.8)	25 (24.3)	11 (22.9)	
Positive	115 (76.2)	78 (75.7)	37 (77.1)	
Hepatitis C, no.(%)				1.000
Negative	145 (96.0)	99 (96.1)	46 (95.8)	
Positive	6 (4.0)	4 (3.9)	2 (4.2)	
Cirrhosis, no.(%)				0.520
Negative	43 (28.5)	31 (30.1)	12 (25.0)	
Positive	108 (71.5)	72 (69.9)	36 (75.0)	
Tumor grade, no.(%)				0.398
Low-grade tumor	100 (66.2)	71(68.9)	29(60.4)	
High-grade tumor	51(33.8)	32 (31.0)	19(39.6)	

*AFP* alpha-fetoprotein, *ALT* alanine aminotransferase, *AST* aspartate aminotransferase, *GGT* gamma-glutamyltransferase

**Table 2:** Baseline clinical characteristics of the high and low Ki-67 expression in training and validation cohort

	Training (n = 103)				Validation (n = 48)			
	High Ki-67 (n = 80)	Low Ki-67 (n = 23)	*P* values		High Ki-67 (n = 32)		Low Ki-67 (n = 16)	*P* values
Age (years), median (IQR)	61.0 (49.0, 67.5)	64.0 (51.3, 70.5)	0.292		54.5 (49.5, 62.0)		57.5.4 (52.5, 70.5)	0.116
Gender, no.(%)			0.721					1.000
Male	62 (77.5)	17 (73.9)			27 (84.4)		13 (81.3)	
Female	18 (22.5)	6 (26.1)			5 (15.6)		3 (18.7)	
ALT,(U/L), median(IQR)	34.3 (21.1, 46.5)	25.0 (19.9, 38.8)	0.207		34.4 (16.0, 35.9)		49.3 (18.3, 73.1)	0.246
AST, (U/L), median(IQR)	33.1 (24.8, 43.3)	30.0 (24.5, 42.5)	0.553		32.1 (21.9, 35.6)		44.1 (23.7, 65.4)	0.341
GGT, (U/L), median(IQR)	49.7 (30.7, 109.8)	46.1 (30.2, 66.0)	0.303		58.7 (16.0, 80.6)		36.8 (27.0, 179.5)	0.974
AFP, (µg/L), median(IQR)	44.4 (4.5, 946.8)	3.3 (1.9, 15.4)	0.000		55.7(2.5, 199.3)		3.8 (2.1, 19.5)	0.006
AFP group.no(%)			0.000					0.026
≤ 20 µg/L	35 (43.8)	19 (82.6)			13 (40.6)		12 (75.0)	
20–400 µg/L	18 (22.5)	3 (13.1)			10 (31.3)		3 (18.8)	
> 400 µg/L	27 (33.7)	1 (4.3)			9 (28.1)		1 (6.2)	
Hepatitis B, no.(%)			0.003					1.000
Negative	16 (20.0)	9 (39.1)			7 (21.9)		4 (25.0)	
Positive	64 (80.0)	14 (60.9)			25 (78.1)		12 (75.0)	
Hepatitis C, no.(%)			0.573					
Negative	76 (95.0)	23 (100)			31 (96.9)		15 (93.8)	1.000
Positive	4 (5.0)	0 (0)			1 (3.1)		1 (6.2)	
Cirrhosis, no.(%)			0.114					0.499
Negative	21 (26.3)	10 (43.5)			7 (21.9)		5 (31.3)	
Positive	59 (73.7)	13 (56.5)			25 (78.1)		11 (68.7)	
Tumor grade, no.(%)			0.040					0.040
Low-grade tumor	45 (56.3)	19 (82.6)			21(65.6)		15 (93.7)	
High-grade tumor	35 (43.7)	4 (17.4)			11 (34.4)		1(6.3)	

*AFP* alpha-fetoprotein, *ALT* alanine aminotransferase, *AST* aspartate aminotransferase, *GGT* gamma-glutamyltransferase

## Features selection and radiomics model development

**Fig. 1:**
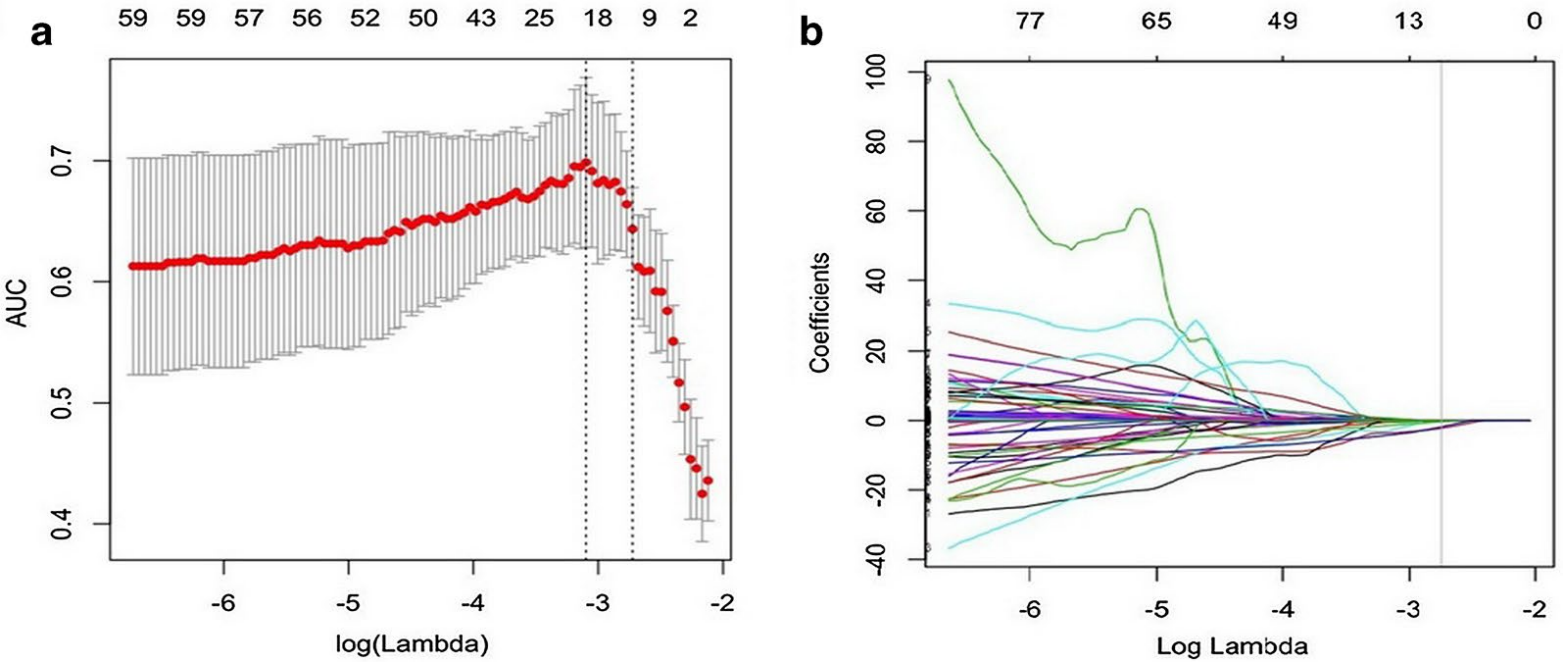
Feature selection using the least absolute shrinkage and selection operator (LASSO) logistic regression in AP radiomics model. **a** Tuning parameter (λ) selection in the LASSO model used 5-fold cross-validation. Dotted vertical lines were drawn at the optimal values by using the minimum criteria and the 1 standard error of the minimum criteria (the 1-SE criteria). A λ value of 0.045, with log (λ), − 2.725 was chosen (1-SE criteria). **b** Vertical line was drawn at the value selected, where optimal λ resulted in 12 nonzero coefficients

No statistically significant difference was found between the inter-observer or between the intra-observer (*P* values ranged from 0.691 to 0.815, 0.755 to 0.891). Of texture features, for AP, HBP, and T2W radiomics models, 1300 features were respectively reduced to 12 (Fig. 1a, b), 6, and 12 potential predictors in 103 patients of the training cohort. For VP images, no valuable features were selected by the LASSO regression analysis. Rad-score was calculated for each patient by using the linear combination of selected features multiplied by their respective coefficients. These features were presented in the Rad-score calculation formula (Additional file 2).

### **Comparison of predictive performance among radiomics models in training and validation cohorts**

**Fig. 2:**
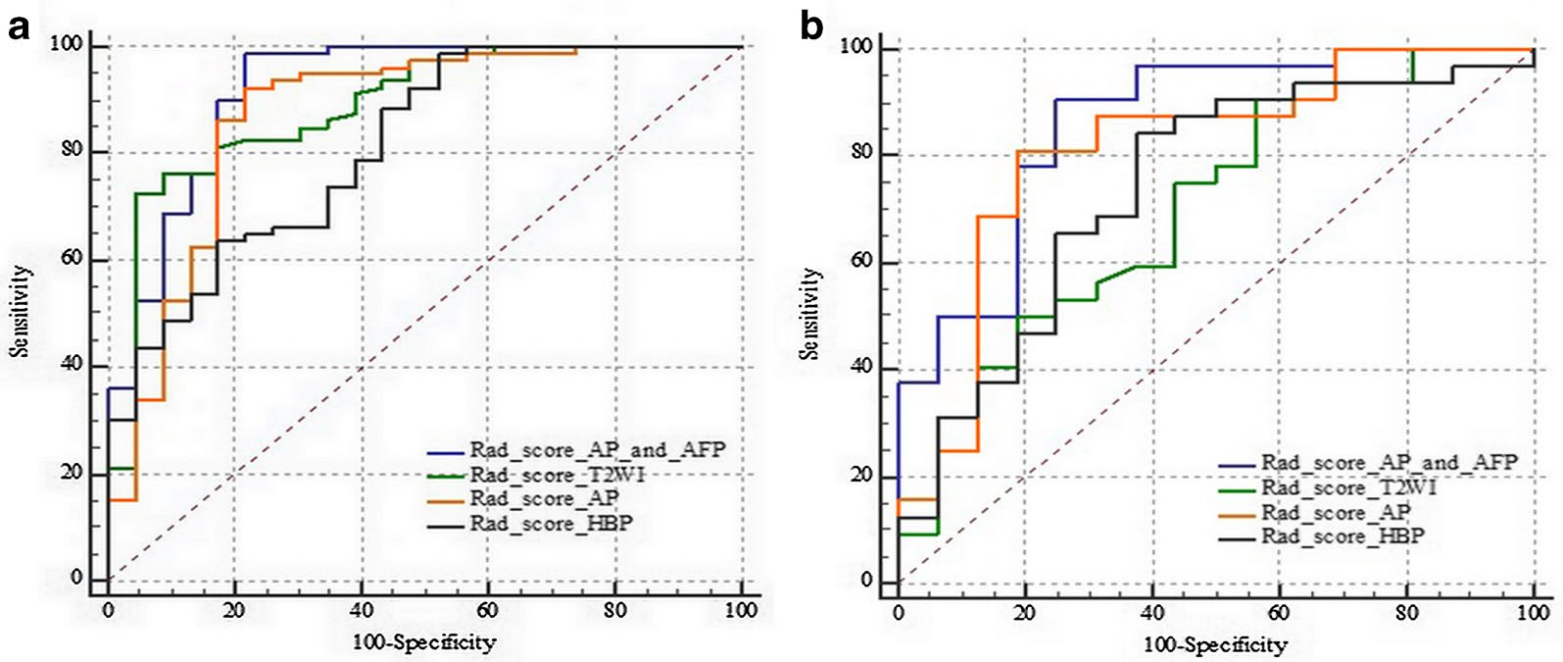
ROC curves for the radiomics model in predicting Ki-67 expression in the training and validation cohort, respectively. **a** ROC curve in training cohort. **b** ROC curve in validation cohort

**Fig. 3:**
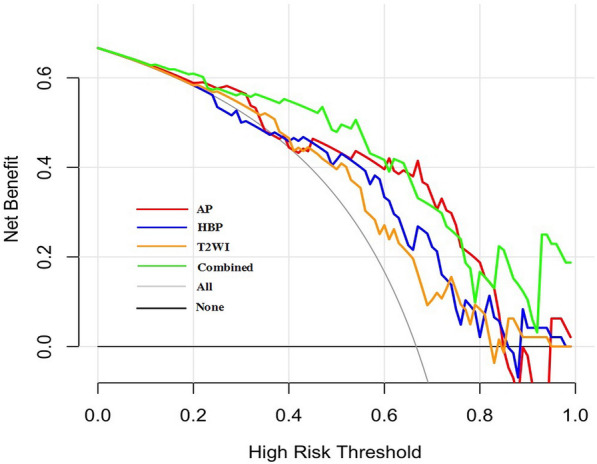
Decision curve analysis of the AP, HBP, T2W radiomics model and combined radiomics model in the validation cohort. The red line, blue line, yellow line, and green line represent the AP, HBP, T2W and the combined radiomics model, respectively. The combined model includes AP Rad-score and serum AFP level. The curve of AP radiomics model was generally higher than that of HBP and T2W radiomics model. Decision curve shows that at a range threshold probability of 30-60 %, the combined model is optimal decision-making strategy to add the net benefit compared with AP radiomics model only

**Fig. 4:**
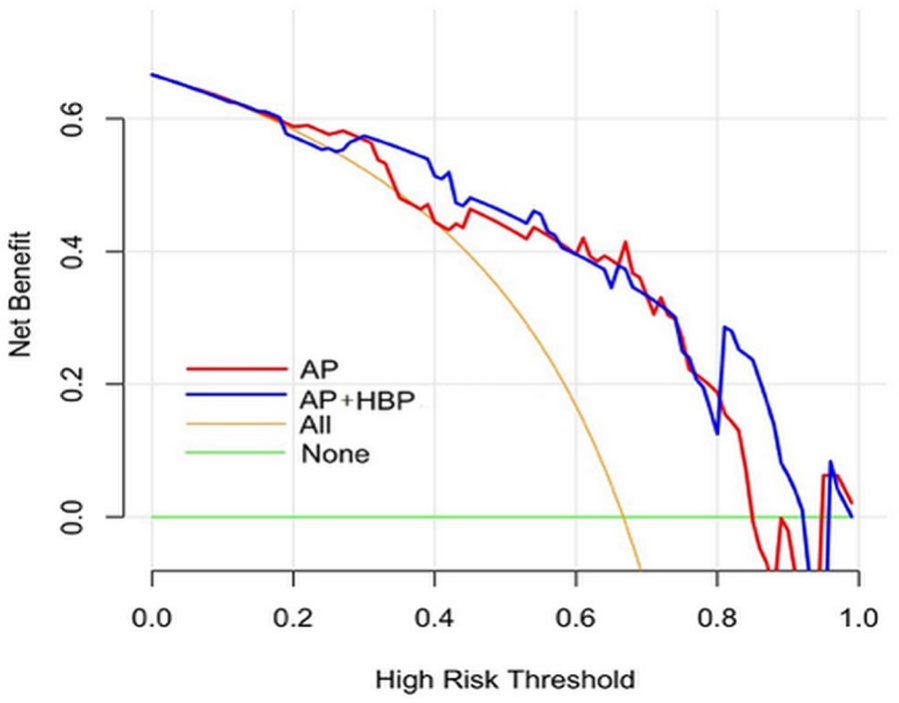
Decision curve analysis of the AP, combined AP and HBP radiomics model in validation cohort. The red line and blue line represent the AP, and combined AP and HBP radiomics model. The decision curve shows that combined AP and HBP radiomics model does not result in extra significant benefits compared with AP radiomics model in validation cohort

The AUC values, sensitivity, specificity, and accuracy of the AP, HBP, T2W, combined AP and HBP radiomics model in predicting Ki-67 expression in training and validation cohort were in Table 3; Fig. 2. Delong test showed that there was no significant difference in AUC values among AP, HBP, combined AP and HBP, and T2W radiomics models. DCA showed that the curve of AP was generally higher than HBP and T2W radiomics models (Fig. 3), and combined AP and HBP radiomics model did not result in significantly extra benefits compared with the AP radiomics model only (Fig. 4).

**Table 3:** Comparison of the predictive performance of the five models in predicting Ki-67 expression

Models	AUC (95% CI)	Sensitivity (%)	Specificity (%)	Accuracy (%)	PPV (%)	NPV (%)
*AP radiomics model*						
Training (n = 103)	0.873 (79.3–93.0)	92.5 (74/80)	78.3 (18/23)	89.3 (92/103)	93.7 (74/79)	75.0 (18/24)
Validation (n = 48)	0.813 (67.4–91.1)	81.3 (26/32)	81.3 (13/16)	81.3 (39/48)	89.7 (26/29)	68.4 (13/19)
Total (n = 151)	0.837 (76.8 89.2)	90.2 (101/112)	69.2 (27/39)	91.4 (138/151)	89.4 (101/113)	71.1 (27/38)
*HBP radiomics mode*l						
Training (n = 103)	0.813 (72.4–88.3)	98.8 (79/80)	47.8 (11/23)	87.4 (90/103)	86.8 (79/91)	91.7 (11/12)
Validation (n = 48)	0.740 (59.3–85.6)	84.4 (27/32)	62.5 (10/16)	89.6 (43/48)	81.8 (27/33)	66.7 (10/15)
Total (n = 151)	0.793 (72.0-85.5)	87.5 (98/112)	59.0 (23/39)	80.1 (121/151)	86.0 (98/114)	62.2 (23/37)
*T2W radiomics model*						
Training (n = 103)	0.889 (81.2–94.4)	72.5 (58/80)	95.7 (22/23)	77.7 (80/103)	98.3 (58/59)	50.0 (22/44)
Validation (n = 48)	0.698 (54.9–82.2)	90.6 (29/32)	43.8 (7/16)	75.0 (36/48)	76.3 (29/38)	70.0 (7/10)
Total (n = 151)	0.823 (75.2, 88.0)	67.9 (76/112)	68.4 (27/39)	68.2 (103/151)	86.4 (76/88)	42.9 (27/63)
*Combined AP and HBP radiomics model*						
Training (n = 103)	0.880 (0.802–0.936)	86.2 (69/80)	82.6 (19/23)	70.9 (73/103)	78.4 (69/88)	26.7 (4/15)
Validation (n = 48)	0.799 (0.658–0.901)	75.0 (24/32)	75.0 (12/16)	75.0 (36/48)	85.7 (24/28)	60.0 (12/20)
Total (n = 151)	0.852 (78.5, 90.4)	83.9 (94/112)	76.9 (30/39)	82.1 (124/151)	91.3 (94/103)	62.5 (30/48)
*Combined model*^a^						
Training (n = 103)	0.922 (0.852–0.965)	98.7 (79/80)	78.3 (18/23)	94.2 (97/103)	94.0 (79/84)	94.7 (18/19)
Validation (n = 48)	0.863 (73.3–94.5)	90.6 (29/32)	75.0 (12/16)	85.4 (41/48)	87.9 (29/33)	80.0 (12/15)
Total (n = 151)	0.806 (73.4–86.6)	83.0 (93/112)	64.1 (25/39)	78.1 (118/151)	86.9 (93/107)	56.8 (25/44)

*AP* alpha-fetoprotein, *AUC* area under receiver operating characteristic curve, *HBP* hepatobiliary phase, *NPV* negative predictive value, *PPV* positive predictive value

^a^Combined model includes AP Rad-score and serum AFP level

## Combined model development and validation

**Fig. 5:**
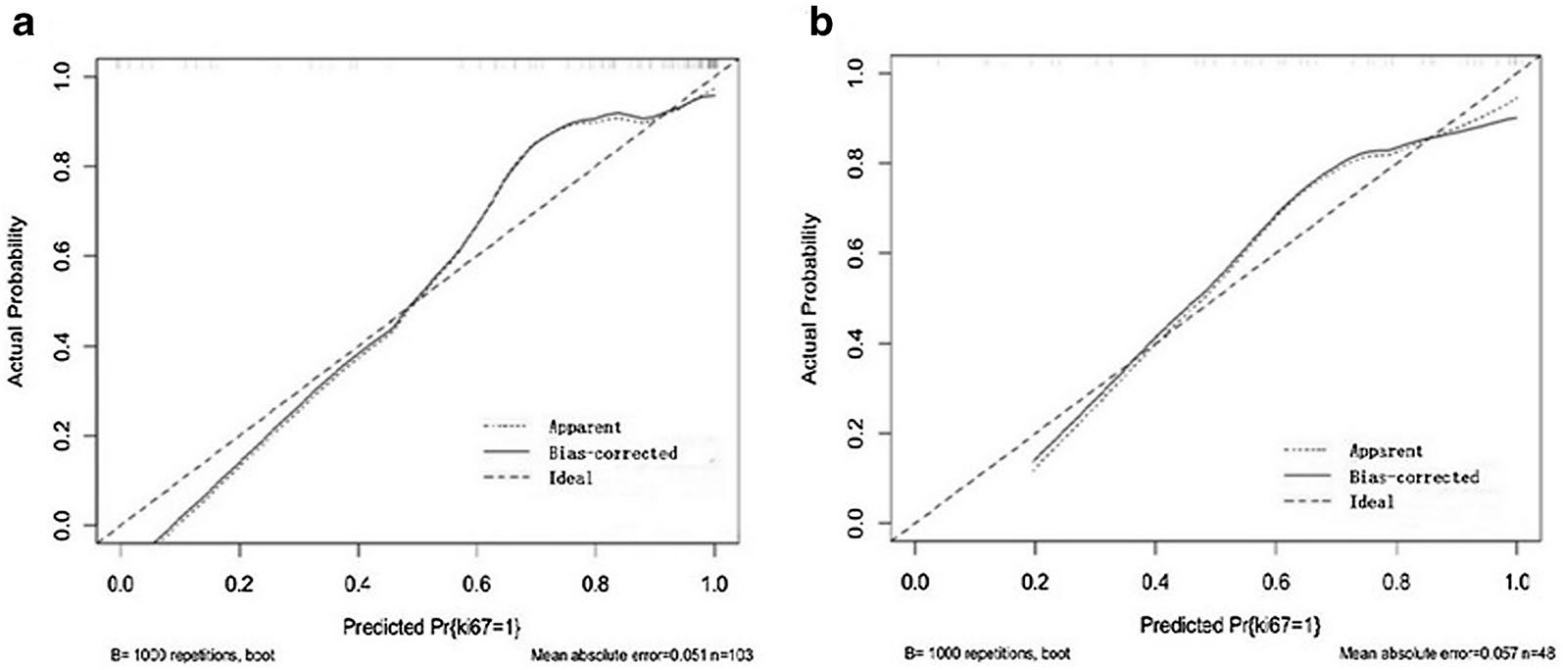
Calibration curve for the combined model in training and validation cohort. **a** Calibration curves for the combined model in training cohort. **b** Calibration curves for the combined model in validation cohort

The multivariate logistic regression analysis showed that only serum AFP level and AP Rad-socre was associated with Ki-67 expression in the training cohort (*P* < 0.05). The combined model was constructed with AP Rad-score and serum AFP level. It yielded an AUC value of 0.922 (95% CI 0.852–0.965) in the training cohort and 0.863 (95% CI 0.733–0.94.5) in the validation cohort (Table 3; Fig. 2). Delong test showed that the AUC value of combined model (0.922, 95% CI 0.852–0.965) was higher than that of AP radiomics model (0.873, 95% CI 0.793–0.930) (*P* = 0.015) in the training cohort. In the validation cohort, the AUC value of combined model (0.863, 95% CI 73.3–94.5) also showed an improved predicting performance in Ki-67 expression over the AUC value of AP radiomics model (0.813, 95% CI 0.674–0.911), despite the non-significant statistical significance (*P* = 0.254). The calibration curves showed a good agreement between predicted and actual events in the training and validation cohorts (Fig. 5a, b). The DCA of the validation cohort revealed that at a range threshold probability of 30–60 %, the combined model is an optimal decision-making strategy to add the net benefit compared with AP radiomics model (Fig. 3).

## Discussion

In this study, we compared the predictive performance of AP, HBP, T2W, and combined AP and HBP radiomics models. Then, we established and validated a combined radiomics model, including AP Rad-score and AFP based on Gd-EOB-DTPA-enhanced MRI for preoperative prediction of Ki-67 expression in patients with HCC. Results showed that the AP radiomics model yielded an incremental performance in predicting Ki-67 expression of HCC over the HBP and T2W radiomics model, and the combined AP and HBP radiomics model does not result in extra benefits compared with the AP radiomics model only. The combined model yielded higher performance with an AUC value of 0.922 (95% CI 0.852–0.965).

As high Ki-67 expression indicates an active status of cell proliferation, which requires more neovascularities for tumor growth. AP images based on enhanced MRI can best demonstrate the information about the neovascularities of tumors. Accordingly, the AP radiomics model added more net benefit in predicting Ki-67 expression of HCC compared with HBP radiomics model. Although a previous study has reported the AP model of Gd-EOB-DTPA-enhanced MRI was inferior, possibly due to artifacts affecting extraction and calculation of textural-based features [26], our study excluded those patients with obvious artifacts caused by transient severe motion (TSM) [27, 28].

Radiomics, including texture analysis and other features, such as shape and intensity [29], is considered to be a potential bridge between medical imaging and personalized medicine [30]. In our study, 41 features most relevant for Ki-67 expression were selected. Among these features, 13 were first-order statistics, 28 were texture features including gray level co-occurrence matrix (GLCM), gray level dependence matrix (GLDM), gray level run length matrix (GLRLM), gray level size zone matrix (GLSZM), and neighboring gray tone dependence matrix (NGTDM). Although some scholars have recently published articles on the same topic of using a radiomics model based on Gd-EOB-DTPA-enhanced MRI to predict Ki-67 expression in HCC [21, 22], there are many differences in details compared with our study. In the study of Li et al. [21], a single slice with the largest proportion of lesion was delineated, and the predictive performance of models were compared only by misclassification rate. In our study, all slices covering the whole tumor were delineated, and, the predictive performance of different models were compared by AUC values, calibration curve and DCA. In the study of Ye et al. [22], a sum of texture signatures derived from AP, PVP, pre-contrast T1W and T2W images was used to predictive Ki-67 expression by multivariate logistical regression, and predictive performance of radiomics model derived from different phases were not be compared. Although, in the study of Ye et al. [22], the C-index (AUC) of the combined model (AUC = 0.936) was approximately equivalent to that in our study—the AUC value of combined model was 0.922 in the training cohort in our study, the study of Ye et al. incorporated a sum of texture signatures derived from multiple phase into one radiomics model, which was cumbersome in clinical practice. Our study developed and compared predictive performance of radiomics models derived from different sequences and phases, including T2W, AP, PVP, and HBP images, then further validated the optimal model for preoperative prediction of Ki-67 expression in HCC, which obtained a good result and would be feasible for clinical practice. Moreover, both of the previous studies lacked the validation cohort to validate whether their models were overfit.

There are several limitations in this study. Firstly, the sample size is still small compared with the number of included variables, especially the sample size of the low Ki-67 expression group, and our validation cohort was from the single institution as the training cohort, which restricted the generalizability of our findings to other institutions or settings. Secondly, our study compared predictive performances of AP, HBP, and T2W radiomics model of the Gd-EOB-DTPA-enhanced MRI for predicting Ki-67 expression of HCC, however, our study did not compare AP radiomics model of Gd-EOB-DTPA-enhanced MRI with Gd-diethylenetriaminepentaacetic acid (Gd-DTPA)-enhanced MRI. Thirdly, there is currently no standardized Ki-67 expression level threshold in HCC, and it may be controversial that we defined 14 % as the cutoff value. In summary, interpreting the complex associations between the biologic processes and radiomics features remains an enormous challenge, although it is in line with the current trend toward precise and personalized medicine.

## Conclusions

Our study established and validated a combined model including AP Rad-score and serum AFP level based on enhanced MRI, for predicting Ki-67 expression in HCC patients. It provides a new non-invasive approach for accurate diagnosis.

## Supplementary Information

**Additional file 1**. MRI protocols and the detailed parameters of the MR sequences.

**Additional file 2**. Rad-score calculation formulae.

## Data Availability

The datasets used or analyzed during the current study available from the corresponding author on reasonable request.

## Abbreviations

AP: arterial phase
GLCM: gray level co-occurrence matrix
GLDM: gray level dependence matrix
GLRLM: gray level run length matrix
GLSZM: gray level size zone matrix
HBP: hepatobiliary phase
NGTDM: neighboring gray tone dependence matrix
Rad-score: radiomics score
