# Supplementary material for: Radiomic analysis of Gd-EOB-DTPA-enhanced MRI predicts Ki-67 expression in hepatocellular carcinoma
Source: BMC Med Imaging. 2021 Jun 15;21:100. doi: 10.1186/s12880-021-00633-0 (PMC8204550; doi:10.1186/s12880-021-00633-0)
Supplement: Supplementary file 1 — Additional file 1. MRI protocols and the detailed parameters of the MR sequences. [file 12880_2021_633_MOESM1_ESM.docx]

**Additional file 1: MRI protocols and the detailed parameters of the MR sequences**

MR images were acquired using a 3.0-T MRI scanner (Magnetom Verio; Siemens Healthcare, Erlangen, Germany) with a 16-channel phase-array coil that covered the whole liver. The MR scan sequences were as follows: 1) a three-dimensional volume interpolated breath-hold examination (3D VIBE) T1-weighted in-phase with time of repetition (TR) = 4.16 ms, time of echo (TE) = 2.58 ms, field of view (FOV) = 26 cm × 32 cm and slice thickness = 5 mm; 2) a 3D VIBE T1-weighted out-phase with TR = 4.16 ms, TE = 1.35 ms , FOV = 26 cm × 32 cm and slice thickness = 5 mm; 3) a respiration-triggered T2-weighted fat-suppression turbo spin-echo with TR = 3, 920-7, 345 ms, TE = 105 ms, FOV = 26 cm × 32 cm and slice thickness = 6 mm; 4) diffusion-weighted imaging (DWI, b = 50, 800 sec/mm^2^) with a free-breathing single-shot echo-planar technique and TR = 5,300 ms, TE = 57 ms, field of view (FOV) = 26 cm × 32 cm and slice thickness = 8 mm.

A food-fast of more than 6 hours and water-fast of more than 4 hours before scanning were required for all patients. The dynamic enhancement was also performed with the 3D VIBE T1-weighted imaging fat saturation sequence with TR = 3.90 ms, TE = 1.89 ms, FOV = 26 cm × 32 cm, and slice thickness =3 mm. A dose of 25 μmol per kg of body weight of Gd-EOB-DTPA (Primovist, Bayer Schering Pharma, AG, Berlin, Germany) was injected as a rapid bolus and was immediately followed by 30 ml saline at a rate of 1 ml/s. The images in arterial phases (AP), portal venous phases (PVP) and transitional phase (TP) were obtained during suspended respiration at 30-35 sec, 65-70 sec, and 180-185 sec, respectively. After injection of Gd-EOB-DTPA, HBP images were obtained at 20 min. AP, PVP, HBP and T2W images were retrieved from a picture archiving and communication system (PACS, Neusoft Version 5.5).
