# Supplementary material for: Radiomic analysis of Gd-EOB-DTPA-enhanced MRI predicts Ki-67 expression in hepatocellular carcinoma
Source: BMC Med Imaging. 2021 Jun 15;21:100. doi: 10.1186/s12880-021-00633-0 (PMC8204550; doi:10.1186/s12880-021-00633-0)
Supplement: Supplementary file 2 — Additional file 2. Rad-score calculation formulae. [file 12880_2021_633_MOESM2_ESM.docx]

**Additional file 2: Rad-score calculation formulae**

**Rad-score_AP_** = (9.54E-03 × log-sigma-1-0-mm-3D _FIRSTORDER_ Skewness ) + ( -3.81E+00 × log-sigma-3-0-mm-3D_GLDM_Dependence_Non_Uniformity_Normalized) + ( -9.24E-01 × log-sigma-4-0-mm-3D_GLCM_MCC) + ( -3.05E-03 × log-sigma-4-0-mm-3D _FIRSTORDER_Inter_quartile_Range) + ( 1.36E-02 × log-sigma-4-0-mm-3D_FIRSTORDER_10Percentile) + (1.14E-02 × log-sigma-5-0-mm-3D_FIRSTORDER_10Percentile) + ( 4.17E-02 × log-sigma-5-0-mm-3D_FIRSTORDER_ Kurtosis) + ( -1.26E-02 × wavelet-LHL_GLRLM_Long_Run_Low_Gray_Level_Emphasis) + ( -2.91E-02 × wavelet-HLH_GLRLM_Long_Run_Low_Gray_Level_Emphasis) + ( -7.09E-06 × wavelet-HLH_NGTDM_ Busyness) + ( -5.09E+00×wavelet-HHH_ FIRSTORDER_ Median) + ( -3.12E+00 × original_GLSZM_Small_Area_Low_Gray_Level_Emphasis)

**Rad-score_HBP_** = (2.49E+00 × log-sigma-2-0-mm-3D _GLCM_ Imc1) + (6.29E-01 × log-sigma-3-0-mm-3D _GLCM_ Imc1) + (1.43E-02 × wavelet-LLH _GLDM_Dependence_Variance) + (-5.93E-01 × wavelet-HHL_GLSZM_Gray_Level_Non_Uniformity_Normalized) + (-2.04E-03 × wavelet-LLL _FIRSTORDER_ Median) + (2.96E-03 × original _GLDM_ Dependence_Variance)

**Rad-score_T2WI_** = (-1.02E+00 × log-sigma-1-0-mm-3D_GLSZM_ Low_Gray_Level_Zone_Emphasis) + (2.12E-01 × log-sigma-2-0-mm-3D_ GLSZM_Small_Area_Emphasis) + (-3.45E-05 × log-sigma-2-0-mm-3D_NGTDM_ Busyness) + (-1.83E-09 × wavelet-LHL_ GLSZM_Large_Area_Low_Gray_Level_Emphasis) + ( 3.27E+00 × wavelet-LHL_ GLCM_ Imc1) + ( -3.32E-01× wavelet-LHL_ NGTDM_ Strength) + (-6.53E-02 × wavelet-LHL_FIRSTORDER_ Mean) + ( -1.91E+00 × wavelet-LHL_ GLSZM_Small_Area_Low_Gray_Level_Emphasis) + ( 2.25E+00 × wavelet-LHL_ GLCM_ Imc1) + ( -1.93E-01 × wavelet-LHL_ FIRSTORDER_ Skewness) +( 4.73E+00 × wavelet-LHL_ GLSZM_Size_Zone_Non_Uniformity_Normalized) + ( -1.47E-06 × wavelet-LLL_ GLSZM_Large_Area_Low_Gray_Level_Emphasis)

**Rad-score_(AP+HPP)_** = (2.35E+00 × log-sigma-2-0-mm-3D _GLCM_ Imc1) + ( 1.86E-02 × wavelet-LLH _ GLDM_ Dependence Variance) + ( -4.19E-01 × wavelet-HHL _GLSZM_ Gray _ Level Non_ Uniformity_Normalized) + ( -1.61E-03 × wavelet-LLL _ FIRSTORDER _ Median) + ( -3.36E+00 × log-sigma-3-0-mm-3D _ GLDM_ Dependence Non_Uniformity_Normalized) + ( 6.81E-03 × log-sigma-4-0-mm-3D _ FIRSTORDER _ 10Percentile) + (1.06E-02 × log-sigma-5-0-mm-3D _FIRSTORDER_ 10Percentile) + ( 1.51E-02 × log-sigma-5-0-mm-3D _ FIRSTORDER _ Kurtosis) + ( -6.19E-03 × wavelet-LHL _GLRLM_ Long_Run_Low_Gray_Level Emphasis) + ( -1.09E-02×wavelet-HLH _GLRLM_Long_Run_Low_Gray_Level_Emphasis) + ( -1.46E+00 × original _ GLSZM_ Small_Area_LowGray_Level_Emphasis)
